# Halogen-doped phosphorescent carbon dots for grayscale patterning

**DOI:** 10.1038/s41377-022-00856-y

**Published:** 2022-05-30

**Authors:** Yanfeng Liu, Mahmoud Al-salihi, Yong Guo, Roman Ziniuk, Songtao Cai, Luwei Wang, Yuan Li, Zhigang Yang, Dengfeng Peng, Kai Xi, Zhongfu An, Xudong Jia, Liwei Liu, Wei Yan, Junle Qu

**Affiliations:** 1 Center for Biomedical Photonics and College of Physics and Optoelectronic Engineering, Key Laboratory of Optoelectronic Devices and Systems of Ministry of Education and Guangdong Province, Shenzhen University, Shenzhen, 518060 China; 2 School of Chemistry and Chemical Engineering, Nanjing University, 163 Xianlin Road, Nanjing, 210023 China; 3 Key Laboratory of Flexible Electronics (KLOFE) and Institute of Advanced Materials (IAM), Nanjing Tech University (NanjingTech), 30 South Puzhu Road, Nanjing, 211816 China

**Keywords:** Imaging and sensing, Quantum dots

## Abstract

Flexible organic materials that exhibit dynamic ultralong room temperature phosphorescence (DURTP) via photoactivation have attracted increasing research interest for their fascinating functions of reversibly writing-reading-erasing graphic information in the form of a long afterglow. However, due to the existence of a nonnegligible activation threshold for the initial exposure dose, the display mode of these materials has thus far been limited to binary patterns. By resorting to halogen element doping of carbon dots (CDs) to enhance intersystem crossing and reduce the activation threshold, we were able to produce, for the first time, a transparent, flexible, and fully programmable DURTP composite film with a reliable grayscale display capacity. Examples of promising applications in UV photography and highly confidential steganography were constructed, partially demonstrating the broad future applications of this material as a programmable platform with a high optical information density.

## Introduction

Light plays an important role in the advanced manufacturing and processing of materials. By introducing photosensitive units and regulating the spatial distribution of the exposure dose, specific patterns and structures have been produced with unprecedented precision and efficiency^1–3^. The developing trend in both optics and materials science therefore has introduced requirements for photoresponsive materials with programmable dynamic performance, namely, the ability to record, reproduce, and erase optical information by phototriggering. To meet this need, materials with photoprogrammable absorption^1,4–7^, fluorescence^6–10^, and even phosphorescence^11–14^ have recently emerged, demonstrating great potential in display, imaging, and optical encryption.

One of these material systems that has drawn particular interest is the group with photoinduced DURTP^15–19^. Taking advantage of the spin-forbidden T_1_ → S_0_ transition, DURTP materials can easily achieve a long afterglow emission observable to the naked eye (*τ* > 50 ms), which not only entirely avoids the excitation background but also provides a large lifetime space for data storage and encryption. Better still, DURTP behaviors have been achieved in organic materials, including polymer composites^18,19^, which are important in terms of having lower toxicity, better machinability, cheaper cost, higher optical transparency, mechanical flexibility, etc. Nevertheless, very few phosphorescent materials with such desirable properties have been reported to date due to difficulties in material design.

**Fig. 1:**
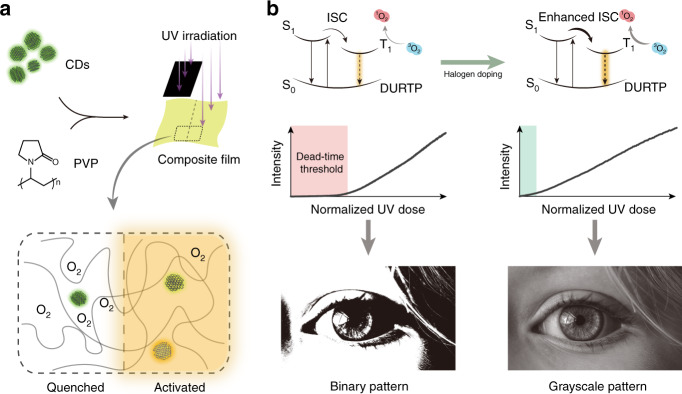
Schematic illustration of the design of halogen-doped CDs composite showing DURTP with grayscale. **a** The photoactivation of DURTP by oxygen regulation within the composite film. **b** Reducing the activation threshold via halogen doping to enable grayscale display

In previous work, we demonstrated for the first time that carbon dots (CDs), a class of emerging phosphorescent nanomaterials^20–22^, could act as reliable DURTP emitters when incorporated with the specific macromolecule material polyvinylpyrrolidone (PVP)^19^. In contrast to the conventional design of static organic phosphorescent materials that emphasized the shielding of environmental oxygen^23–26^, the DURTP in CDs/PVP composites was facilitated by an oxygen-regulating mechanism. Here, PVP acted both as a solid matrix and an oxygen reservoir, simultaneously providing hydrogen bonding fixation that facilitates the DURTP of CDs and triplet oxygen that suppresses its emission. CDs also played a dual role, both as an oxygen-sensitive phosphor and as an oxygen-consuming photosensitizer. An activation process via mask and photolithography could therefore regulate the regional “on/off” switch of DURTP, realizing the manipulation of erasable long afterglow patterns on a transparent flexible film (Fig. 1a). However, although the oxygen-regulated DURTP with CDs/PVP was more than qualified for a binary display (which distinguished only the “on” and “off” states), it became insufficient when considering a grayscale display requirement (which displayed a series of intensities with a gradient). Undoubtedly, this issue has hindered DURTP materials from reaching their full potential in further applications. The crux of the matter lies in the high threshold or “dead time” in DURTP activation. In other words, the response of DURTP intensity to photoactivation occurs with a considerable delay, causing the loss of shadow details in the grayscale pattern.

The challenge commonly faced by almost all oxygen-regulated dynamic phosphorescent systems^12,18,19^ is fundamentally limitation by the insufficient ISC of metal-free dynamic phosphors. First, organic phosphors with low ISC rates are less tolerant to triplet oxygen, showing significant quenching even at a low oxygen concentration^27,28^. Second, since the phosphor also acts as an oxygen-consuming photosensitizer in DURTP, an insufficient yield of triplet excitons should also limit the oxygen consumption rate, providing the same photon dose, which further contributes to the issue. To address that, a straightforward solution would be modifying the preexisting material systems by introducing molecular structures that facilitate ISC, such as lone pair electrons. To date, although certain functional groups, such as nitrogen heterocyclic rings and phosphonates with lone pair electrons, have been introduced to DURTP systems to improve their performance^29,30^, none of them have reported grayscale display capacities.

In addition to introducing lone pair electrons, another strategy commonly used for improving the ISC efficiency is heavy atom substitution/doping, especially by halogen atoms^31,32^. Typically, doping with halogen atoms induces a series of changes in the phosphor known as the “heavy atom effect”, enhancing spin-orbit coupling and consequently facilitating ISC and phosphorescent emission. However, halogen doping might also induce changes in polarity and hydrogen bonds, interfering with phosphor-polymer compatibility. Luckily, the surface of solvothermal-synthesized CDs is highly functionalized with hydrophilic groups^33,34^, which should likely compensate for the change in compatibility induced by halogen doping. Moreover, the possibility of halogen doping in CDs has been previously validated. For instance, Feng’s group reported the synthesis of F-doped phosphorescent CDs by introducing hydrogen fluoride/fluorine in the bottom-up synthesis of CDs^35–37^. These successful precedents have encouraged us to further consider heavy halogen doping as an applicable strategy for enhancing ISC in DURTPs.

In this work, we propose a series of flexible DURTP polymer composites illuminated by halogen-doped CDs with fully programmable emissions. We show that the enhancement of ISC and the suppression of the activation threshold are dependent on the doping degree of CDs and successfully achieve a grayscale display with a composite with the highest halogen content (Fig. 1b). Taking advantage of the high phosphorescence quantum yield, long afterglow emission, grayscale display capacity, and ultrafast UV response of this composite, the applications of DURTP materials in grayscale-based UV photography and steganography have been explored for the first time, demonstrating the broad possibilities for optical applications of this spectacular group of materials.

## Results

**Fig. 2:**
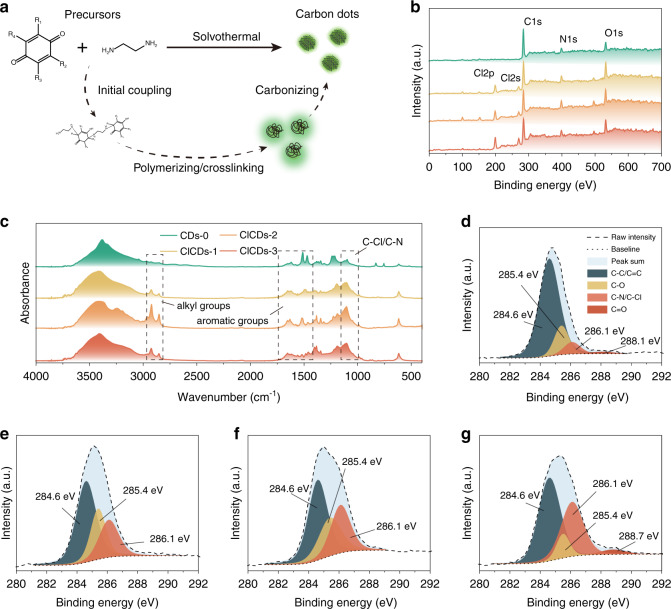
Chemical compositions of CDs with different halogen contents. **a** Schematic illustration of the solvothermal synthesis of CDs from molecular precursors. (For CDs-0, R1 = R2 = R3 = R4 = H; for ClCDs-1, R1 = Cl, R2 = R3 = R4 = H; for ClCDs-2, R1 = R3 = Cl, R2 = R4 = H; for ClCDs-3, R1 = R2 = R3 = R4 = Cl.) **b** XPS survey and elemental compositions of the four CDs. **c** The FT-IR absorbance of the four CDs. Deconvoluted C1s binding energies of CDs-0 (**d**), ClCDs-1 (**e**), ClCDs-2 (**f**), and ClCDs-3 (**g**)

Since the CD material was synthesized by a bottom-up strategy from molecular precursors, the halogen-doping of CDs could be easily achieved by using halogenated precursors in the synthesis (Fig. 2a). Notably, although Br or I atoms may provide a more significant heavy atom effect, their larger atom diameters and lower bonding energies with carbon also cause a higher leaving tendency during the formation of CDs. As a result, low (to zero) contents of these elements were found in the CDs made of iodinated and brominated precursors (Fig. S1). As the second-best option, we synthesized a series of CDs using chlorinated derivatives of p-benzoquinone under solvothermal conditions, which is similar to the previous method we adopted to synthesize halogen-free CDs^19^.

The resultant halogen-doped CDs, ClCDs-1~3, gradually increased in chloride contents from 6.0 to 9.3%, depending on the number of substituents in their precursors (Fig. 2b and Table 1). Compared to their halogen-free cousin CDs-0, the intensified signal at ~1100 cm^−1^ in the FT-IR absorbance of ClCDs-1–3 indicated the presence of C-Cl (Fig. 2c and Table S1), which was also validated by the increase in the C-Cl signal intensities in the C1s and Cl2p binding energies of the CDs (Fig. 2d–g). Moreover, the deviation in the Cl2p binding energy suggested that Cl atoms were binding with both sp2- and sp3-hybridized C in the halogen-doped materials (Fig. S2). Despite the differences in the chemical compositions, all four CDs showed very similar morphologies with average diameters ranging from 2.6–2.8 nm (Fig. S3). Stripes with regular intervals of ~0.24 nm (corresponding to the [1 0 0] lattice plane of the graphitic structure) were observed in the HRTEM results, suggesting the crystalline nature of these nanoparticles. The crystalline structure in CDs was also revealed by the X-ray diffraction (XRD) spectra of these materials. As shown in Fig. S4, broad peaks at 2*θ* = 23° and bumps at 2*θ* = 45° were detected, corresponding to the [0 0 2] and [1 0 0] lattice planes of nanographite^38,39^.

**Table 1:** Elemental compositions of the four CDs determined by an XPS survey

CDs	C content (At%)	N content (At%)	O content (At%)	Cl content (At%)
CDs-0	80.64%	9.08%	10.01%	0.27% (noise)
ClCDs-1	68.06%	8.66%	17.29%	5.98%
ClCDs-2	65.94%	11.17%	15.44%	7.45%
ClCDs-3	64.62%	10.63%	15.42%	9.33%

**Fig. 3:**
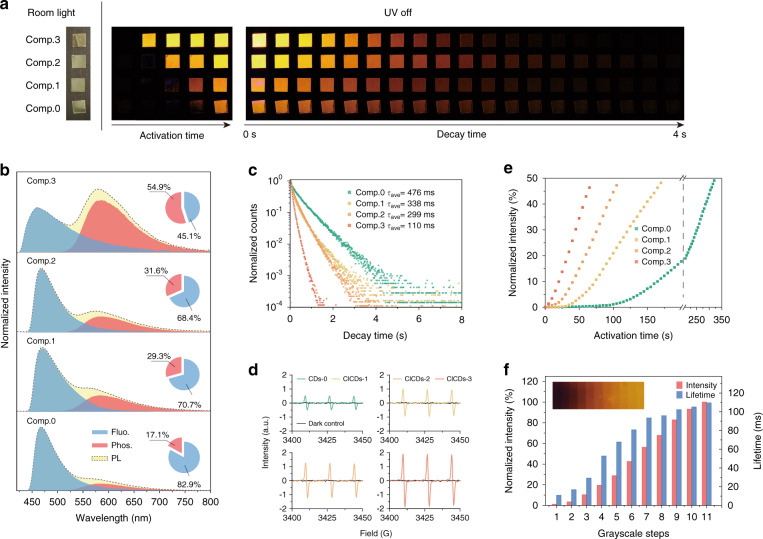
DURTP characteristics of CDs/PVP composites with different halogen contents. **a** Photographs depicting the photoactivation and decay features of four CDs/PVP composites. **b** The phosphorescent component in the total photoluminescence of four CDs/PVP composites. **c** The lifetime decay profiles of the four CDs/PVP composites (activated). **d** ESR signals of TEMP-captured ^1^O_2_ at 0 min (dark control) and 2 min after UV irradiation in the presence of the four different CDs (20 μg/ml). **e** Photoactivation profiles of the four CDs/PVP composites. **f** The changes in the lifetimes and intensities of composite 3 corresponding to the effective UV dose at different grayscale steps

In terms of their optical features, CDs-0 and the three halogen-doped CDs showed similar absorption and fluorescence emission curves in ethanol (Fig. S5), suggesting that the Cl doping did not cause severe changes in the excited state energy levels of the CDs. To further facilitate DURTP emission, the four CDs were embedded in solid PVP matrices through solution blending and film casting. The nonradiative relaxation of the triplet excited states was thus sufficiently suppressed through hydrogen bond fixation between the polymer and the hydrophilic functional groups of CDs. Under a common atmosphere environment, the CDs/PVP composite films showed no significant long afterglow when initially irradiated by a short pulse of UV excitation (400 nm, 10 mW cm^−2^, 20 ms). This stage corresponds to the “off” state of the DURTP since the environmental oxygen molecules have permeated the polymer films and caused the quenching of triplet excitons of CDs. With a prolonged irradiation time, the orange-colored phosphorescent emission of the CDs/PVP films (denoted as composite 0–3 hereafter) becomes activated, showing seconds of afterglow when the excitation switches off (Fig. 3a). This stage corresponds to the “on” state of the DURTP, where the permeated oxygen has been efficiently removed through photochemical reactions, allowing phosphorescent emission from the triplet excited states of CDs (Fig. 1). From this point, the DURTP emission of the films could be readily evoked by short irradiation pulses before the environmental oxygen again permeated the film through molecular thermal motion. At room temperature (25 °C) and under a common atmosphere, the DURTP intensities slowly decreased to nondetectable intensities in ~2.5 h (Fig. S6). Naturally, the deactivation of DURTP by oxygen could be accelerated by heating the composite films under a common atmosphere: typically, the fully activated DURTP could be entirely deactivated by baking the samples at 120 °C for 10 min. For all four CDs/PVP composites, such activation and deactivation of DURTP have proven to be highly reversible, as ten cycles of on-off switching did not induce a significant change in their activated DURTP intensities (Fig. S7).

The emission spectra and lifetimes of the four composites confirmed that the doping of Cl played an important role in promoting the ISC in excited states: as the doping of Cl in CDs increased, the percentage of the phosphorescent component in the activated steady-state photoluminescence emission increased markedly from 17.1% (composite 0) to 54.9% (composite 3) (Fig. 3b), while the lifetime of the activated DURTP gradually decreased from 472 to 110 ms (Figs. 3c and S8 and Table S2), in accordance with the typical heavy-atom effect. By measuring the fluorescence quantum yields of the deactivated composites and multiplying them with the *I*_Phos._/*I*_Fluo._ ratio, we were able to calculate the phosphorescence quantum yields of these materials (Fig. S9 and Table S3). Notably, the overall phosphorescence quantum yield (*λ*_ex_ = 400 nm) of composite 3 was 2.93%, which was comparable to some molecular long afterglow phosphors in a similar wavelength range^32,40^. Moreover, the phosphorescence quantum yields of composites 0–2 were 0.94%, 1.61%, and 1.93%, respectively, clearly showing an increasing tendency as the halogen atom content increased.

The enhancement of ISC in CDs should change their photoactivation behavior in two ways. First, the enhanced radiative transition of T_1_ → S_0_ increased the tolerance of phosphorescent emission to the environmental oxygen. In other words, the initial activation of DURTP occurred earlier with a higher oxygen content in the composite film, thus shortening the dead-time threshold. On the other hand, the increased population of triplet excited states led to higher photodynamic conversion capacities, increasing the speed of DURTP activation. Here, the singlet oxygen productivities of all four CDs were measured by electron spin resonance spectroscopy. As shown in Fig. 3d, the halogen-doped CDs, especially ClCDs-3, featured a highly improved photodynamic efficiency (~4.1 times that of CDs-0). As a result, much faster DURTP activation kinetics were observed in the halogen-doped composites (Fig. 3e). First, the values of the characteristic parameter *t*_1/2_ (that is, the time required to reach the half-maximal DURTP intensity under a certain power density) for composites 1–3 at 0.1 mW cm^−2^ were 175, 110, and 71 s, respectively, which were all significantly shorter than that for composite 0 (325 s). Second, the activation threshold (determined by the intercept of the activating curve on the *X*-axis, see Fig. S10) clearly decreased with an increasing Cl content in the materials. Here, we defined the relative threshold *R*_th_ as the ratio of the deadtime to *t*_1/2_. The dramatic decreases in the deadtime from 123 to 6.7 s and in *R*_th_ from 0.38 to 0.09 unambiguously confirmed that the halogen-doping strategy improved the performance of the CDs/PVP composites in terms of both the response speed and the threshold.

Based on this knowledge, we further tested the applicability of the composite as a grayscale-based display medium. As the most promising candidate with the smallest threshold, composite 3 was studied at different stages of activation. Figure S11 depicts the gradually increasing phosphorescence intensities and lifetimes of this composite during activation. In summary, an 84-fold enhancement in intensity and ten-fold enhancement in lifetime (from 11 to 110 ms) occurred during the activation of DURTP. According to these results, we envisaged that a grayscale-based display of DURTP could be manipulated in the range of 0–20 mJ cm^−2^. Here, we applied a transparent mask printed with a tailored ITE grayscale chart (Fig. S12) to regulate the light dose and create a phosphorescent grayscale step-chart on a flexible film of composite 3. The relationship between the normalized intensities/lifetimes and the UV light dose at different grayscale steps is given in Fig. 3f. After exposing the film to 20 mJ cm^−2^ UV, a series of grids with intensities increasing stepwise were obtained, demonstrating the potential of this material for grayscale display (Fig. 3f, inserted). In contrast, a composite 0 film displayed only part of the grayscale gradient due to its high activating threshold (Fig. S13).

## Discussion

**Fig. 4:**
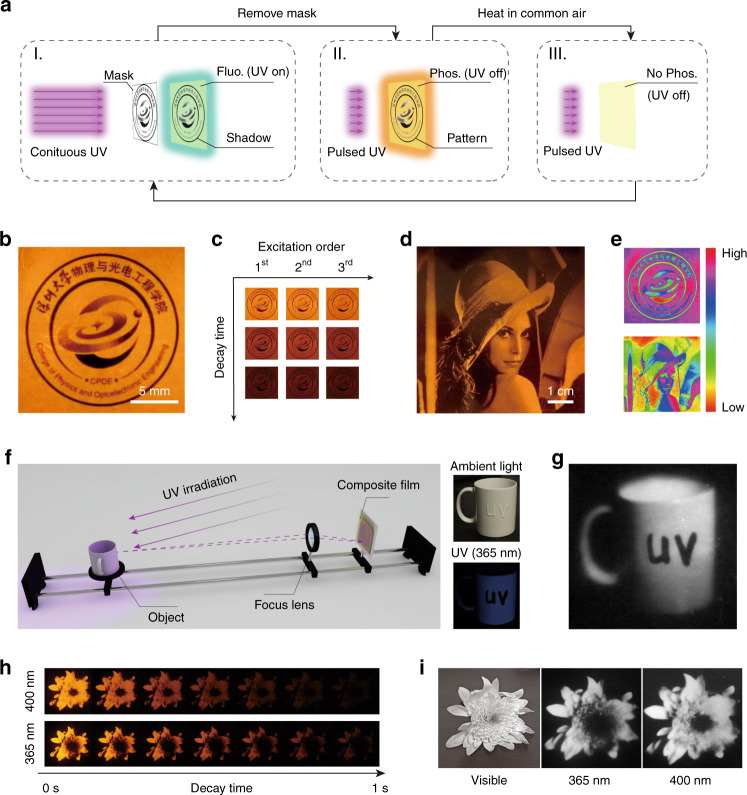
The processing of halogen-doped CDs composite for grayscale patterning by mask and lens. **a** Schematic illustration of the photopatterning (I), reproduction (II) and thermal reset (III) procedures of creating grayscale graphs. **b** Photograph of a grayscale icon created by mask and photolithography. **c** Photographs of the patterned icon with repeated excitations. **d** Photograph of a grayscale portrait created by mask and photolithography. **e** The grayscale distribution of **b** and **d** depicted in colored LUT. **f** Scheme for UV photography with a CDs composite film. **g** Grayscale UV image (inverted for display) reproduced from the DURTP. **h** Decay of the DURTP long afterglow patterns of a daisy flower captured by UV photography. **i** Comparison of the grayscale photographs captured in the visible range (using a commercial camera), 365 and 400 nm (captured with composite 3 through a focal lens)

The grayscale display capacity and reversible DURTP functions of halogen-doped CDs composites have enabled a number of unique optical applications. Here, a repeatedly editable DURTP tag with designable print-on-demand grayscale patterns was developed by utilizing the photoactivation and thermal-deactivation behaviors of DURTP. As illustrated in Fig. 4a and Movie S1, the designed grayscale DURTP patterns could be created through a photolithography process and erased through heating for multiple cycles. With a proper mask, the resolution of such DURTP patterns could reach ~35 μm, corresponding to over 724 dpi in the display (Fig. S14). After photowriting and removing the mask, the grayscale pattern could be readily reproduced by exposing the film to a short UV pulse. Judging from the excitation dynamics and activation threshold of composite 3 (Fig. S15), a total UV dose in the range of 0.02–0.65 mJ cm^−2^ (2–65 ms at 10 mW cm^−2^) is considered to be suitable for reproducing the patterns. As shown in Fig. 4b–e, the film of composite 3 provided an excellent grayscale distribution that enabled the display of an intensity gradient in both icons and portraits. Notably, the composite film itself was highly flexible and could be attached to a curved surface (Fig. S16), further broadening its potential application in print-on-demand temporary tags integrating optical anti-counterfeiting functions^18,19^.

UV photography has long been recognized as a powerful tool for optical diagnosis and biological studies by providing imaging results beyond the visible range^41,42^. Considering the low activation dose and grayscale display capacity of composite 3, we envisaged that this material could also function as a UV photographic film to directly capture UV images from object reflections. To verify this idea, a simplified imaging system mimicking the structure of the film camera was built, as illustrated in Fig. 4f. The UV-reflecting object (a white porcelain mug cup) partially coated with a UV-absorbing sunscreen was placed in front of a convex lens against the UV-sensitive film (composite 3). With a large object distance (≫2 focal lengths) and a small image distance (≪2 focal lengths), a miniature and inverted image of the object occurred on the film under UV irradiation. With sufficient exposure, an image of the object could be captured by the UV-sensitive film and then reproduced with pulsed excitation. In contrast to what was shown in the visible-light photo (Fig. 4f, inserted), the sunscreen appeared as dark shadows in the UV photography image (Fig. 4g), showing strong UV absorption in these areas.

Many plants have developed UV-absorbing patterns on their flowers to attract pollinating insects with UV color vision^43^. With the same technique demonstrated above, we captured UV photographs of a daisy flower at 365 and 400 nm. Here, two grayscale images were reproduced from the photoinduced long afterglow pattern in the composite film after UV exposure (Fig. 4h, i). In contrast to the grayscale visible image, both UV photographs showed a region of high UV absorbance in the center of the flower where the stamens were mostly located (Fig. 4i). Moreover, in the 365 nm photograph, a dark halo was observed surrounding the center, suggesting the existence of a concentric-circular structure with a UV color gradient.

As discussed above, the DURTP of composite 3 gradually increased both in terms of its intensities and lifetimes upon activation. While the precise reading of emission intensity might be influenced by environmental noise and other issues, luminescence lifetimes are considered highly characteristic and conservative, providing reliable performance in optical encrypting scenarios. Thus, lifetime-based steganography has long been an intriguing topic among the various applications of persistently luminescent materials. However, most previous works have focused on time-gating-based methods^20–22,40,44^, which usually require a large lifetime contrast between the cover message and the real message (for example, ~ns/fluorescence versus ~ms/phosphorescence) and hence have shown limited encryption capacity. In this work, taking advantage of the multiple merits of composite 3, including a fast activation dynamic, a visible long afterglow, and very importantly, a highly manipulatable emission lifetime, we have proposed a new strategy for highly confidential steganography by grayscale DURTP.

**Fig. 5:**
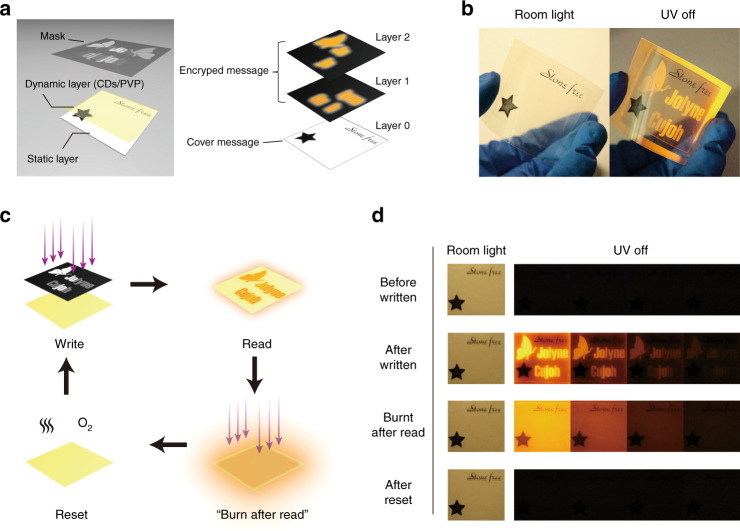
The design of highly confidential steganography based on the grayscale DURTP of halogen-doped CDs composite. **a** The physical and informational structures of the steganographic device. **b** Photographs of the activated steganographic device under room light and after UV light was switched off. Note that the whole device is transparent and highly flexible. **c** Scheme for the cycle of “write/read/burn-after-read/reset” of the dynamic layer. **d** Photographs of the steganographic device at different stages in the encryption cycle

Figure 5a schematically shows our design of a reusable dynamic steganographic device in practice: the film device physically consists of two layers, a static layer with apparent text and a dynamic layer with a latent DURTP pattern printed on demand. Due to the high transparency of the PVP polymer film, the text or patterns on the static layer could be clearly seen under ambient light (Fig. 5b), acting as the cover message in this sense. When performing optical encryption, a piece of the grayscale mask was first applied to write the lifetime-coded secret message on the dynamic layer with UV exposure (Fig. 5c). In this demonstration, the mask contained two types of text patterned with different transmittances, corresponding to two informational layers of graphics with different grayscale values and lifetimes. Then, the mask was removed, and a short UV pulse was used to read the hidden message in the form of afterglow (Fig. 5b, c). To further improve the confidentiality of this steganographic device, the sheet was then overexposed via continuous UV irradiation to exert a “burn after reading” function. Finally, simply by heating in a common atmosphere, the device could be reset to the initial state for the next use (Fig. 5c, d).

**Fig. 6:**
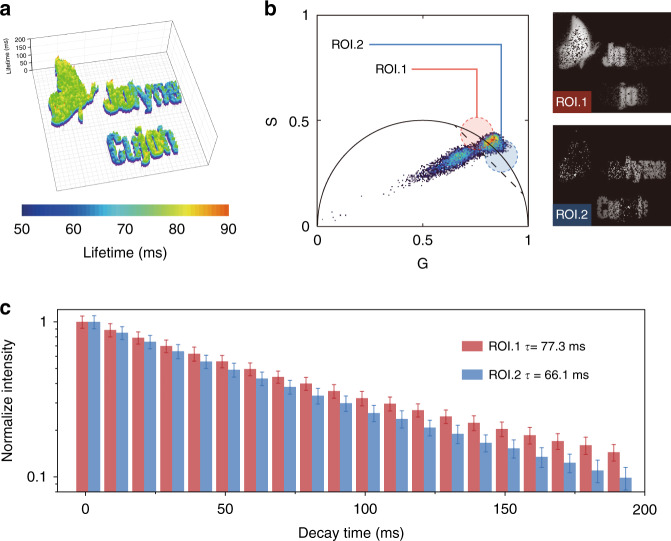
Multilayered message decrypted by lifetime imaging. **a** Lifetime mapping of the steganographic pattern. **b** Phasor analysis of the steganographic pattern. **c** Average decay profiles of the pixels in two different ROIs

To take full advantage of the lifetime gradient of this device, we introduced lifetime imaging instead of time gating in the “read” procedure to extract the encrypted graphical message. Figure S17 illustrates the fundamentals of the afterglow lifetime imaging setups. In short, a pulsed diode laser device was connected to an ICCD camera with an external trigger to control the image capture at different delayed times after the excitation pulse. As a result, the pixelwise decay profile was obtained and analyzed. After denoising and thresholding, the mapping results clearly showed distinguishable lifetimes (66 ms/77 ms) in the two parts of the DURTP graphics (Fig. 6a, b). Notably, the lifetime difference (11 ms) was only ~10% of the fully activated lifetime value of composite 3, which suggested high potential for increasing the encryption capacity with this lifetime-coded steganography design.

Finally, to avoid the complex denoising process and further simplify the decryption process, we also introduced a phasor analysis method to resolve lifetime information in the frequency domain^45^. Here, the phasor plot diagram was drawn with a MATLAB program we previously developed^46^. As depicted in Fig. S18, the phasor plot results were clearly divided into two clusters corresponding to background noise and signal. The signal spots were distributed along the semicircle curve, corresponding to the PL signal emitted from patterned areas featuring quasi-monoexponential decay. Meanwhile, the background noise induced by intramembrane refraction, reflection, and scattering distributed along a vector pointed from (0, 0) to the signal cluster, indicating the complexity of their origins. Further examining the signal cluster showed that the two layers of the encrypted graphic message could be well separated by selecting different regions along the curve. ROIs 1 and 2 correspond to pixels with different PL lifetimes, which were facilely resolved into two images (Fig. 6c). Importantly, since no curve fitting is required in the phasor analysis^47^, the amount of calculation required to resolve the encrypted message is greatly reduced, thus allowing near-real-time decryption of the message. Accordingly, we envision that the combination of grayscale-based lifetime encryption and phasor analysis has the potential to become a standard solution for DURTP-based steganography design in the future.

In summary, in this work, we proposed a facile strategy to achieve programmable DURTP with a low activation threshold by enhancing its ISC through halogen doping in CDs. On that basis, we have further contrived the demonstration of grayscale-based patterning, UV photography and steganography in flexible films, showing the promising potential of this material for advanced optical applications. Overall, we believe that our findings in this work reveal a new direction for the applications of DURTP materials and contribute special insights into the rational synthesis, luminescence and optical applications of CDs.

## Materials and methods

### Chemicals and materials

p-Benzoquinone (LR. 99%), 2-chloro-1,4-benzoquinone (LR, 95%), 2,5-dichloro-1,4-benzoquinone (LR, 98%), p-chloranil (LR, 98%), p-bromanil (LR, 98%), NaI and KI (GR, 99.5%), ethylenediamine monohydrate (LR, 98%) and PVP (K88~96, Mw = 1300k) were purchased from Aladdin Chemistry Co., Ltd. (Shanghai, China). Dichloromethane (AR), methanol (AR), and ethanol (AR) were purchased from Titanchem Co., Ltd. (Shanghai, China). Double-distilled water was used throughout this work. All reagents were used as received if not otherwise specified.

p-Iodanil was synthesized according to a previous reference^48^ with modification: briefly, 3 g (7 mmol) of powdered p-bromanil was treated with KI (2.3 g, 14 mmol) in ethanol (30 ml) and refluxed for 2 h. After being filtered, the solid was further treated with 2.1 g NaI (14 mmol) in ethanol (30 ml) and refluxed for another 2 h. The dark-brown product was isolated by filtration and recrystallized in ethyl acetate (mp 279–280 °C).

### Apparatus and characterization

The infrared absorbance spectra were measured on a Thermo Scientific Nicolet iS5 FT-IR spectrometer (Thermo, US), and the UV–vis absorption spectra were measured with a Cintra 2020 spectrometer (GBC, Australia). X-ray photoelectron spectroscopy of CDs was performed on a PHI 5000 Versa Probe machine (UlVAC-PHI, Japan). The transmission electron microscopy images of the CDs were captured on an F-200 device (JEOL, Japan), and the powder XRD patterns were measured on a MiniFlex600 device (Rigaku, Japan) with a Cu target (*λ* = 1.5405 Å).

For photoluminescence characterization, the emission spectra and lifetimes were measured on an FLS-1000 spectrometer, while the quantum yields were measured with a C9920-02G absolute quantum yield measurement system (Hamamatsu Photonics, Japan).

For lifetime imaging, a wide-field time-resolved system consisting of a pulsed laser, ICCD camera, and optical configurations (camera lens and long-ass filter) was adopted. The emission from the sample was captured by an ICCD, model DH312T-18U-03, from Andor Technology.

### Synthesis of the CDs

The synthesis of CDs was adopted from previous literature with some modification^19^. Two types of molecular precursors, the p-benzoquinone derivatives and ethylenediamine monohydrate, were used together to synthesize CDs through a one-pot solvothermal procedure. In general, 0.5 mmol of p-benzoquinone derivatives were dispersed in 100 ml of ethanol by bath ultrasonication. After adding 1 mmol of ethylenediamine monohydrate (81 μl), the dispersion quickly turned to dark hazel. The mixture was sealed in a stainless-steel autoclave and heated to 160 °C for 12 h before cooling to room temperature. The raw product was concentrated by rotary evaporation, dialyzed against water (Mw_cutoff_ = 2000 D, 24 h), dried under reduced pressure and purified via silica column chromatography (eluent: 5–15% methanol in dichloromethane) for further use.

### Fabrication of the CDs/PVP composite films

Typically, to fabricate a CDs/PVP composite film, 1 g of vacuum-dried PVP was dissolved in 40 ml of double-distilled water under mild agitation at 50 °C to form a homogeneous solution. Then, 5 mg of CDs dissolved in 1 ml of methanol was quickly added by injection. The mixture was kept at 65 °C under agitation for 60 min to remove methanol and prevent the formation of visible bubbles.

Afterward, the viscous solution was poured onto a 20 cm × 20 cm square polystyrene petri dish and kept at 30 °C in the atmosphere overnight to remove the solvent. (Note that the heating device should be leveled in advance to obtain a high-quality film with uniform thickness.) After shaping, the composite film was gently peeled from the dish and further dried at 120 °C and 0.1 mbar for 2 h. The resultant film (50 μm in thickness) was then sealed with 50 μm PET via lamination for further use.

### Lifetime imaging and phasor analysis

The lifetime imaging system was set up as illustrated in Fig. S15. A pulsed UV beam with a 20 ms width (2 mW cm^−2^) was triggered at a 1 Hz frequency to excite the activated sample with a designed DURTP pattern. Here, the ICCD was triggered through a BNC cable with a TTL signal synchronized with the laser beam. The emission signal was collected in the same direction as the excitation beam by a camera lens combined with a longpass filter. The captured images were analyzed by both lifetime mapping and phasor analysis methods.

For lifetime mapping, the sequential intensities of each pixel were extracted and fitted with an exponential decay model to give their lifetime values. For phasor analysis, the decay profile of each pixel was processed in the frequency domain according to the following calculations:

$$G\left( \omega \right) = \frac{1}{I}\cdot \mathop {\sum }\limits_{k = 1}^N C_k\cos \omega t_k$$

$$S\left( \omega \right) = \frac{1}{I}\cdot \mathop {\sum }\limits_{k = 1}^{{{\mathrm{N}}}} C_k\sin \omega t_k$$

where (*G*, *S*) are the phasor plot coordinates of a given pixel, *ω* is the angular frequency of the pulsed laser, *C*_*k*_ is the emission intensity at a certain time (channel *k*) after excitation, *I* is the summation of *C*_*k*_, *N* is the total number of channels, and *t*_*k*_ is the width of channels in time.

## Supplementary information

Supporting information

Supporting information
