# Supplementary material for: Halogen-doped phosphorescent carbon dots for grayscale patterning
Source: Light Sci Appl. 2022 May 30;11:163. doi: 10.1038/s41377-022-00856-y (PMC9151715; doi:10.1038/s41377-022-00856-y)
Supplement: Supplementary file 1 — Supporting information [file 41377_2022_856_MOESM1_ESM.docx]

**Supporting information for**

**Halogen-doped phosphorescent carbon dots for grayscale patterning**

**Yanfeng Liu^1^, Mahmoud Al-salihi^1^, Yong Guo^1^, Roman Ziniuk^1^, Songtao Cai^1^, Luwei Wang^1^, Yuan Li^1^, Zhigang Yang^1^, Dengfeng Peng^1^, Kai Xi^2^, Zhongfu An^3^, Xudong Jia^2^, Liwei Liu^1^, Wei Yan^1^*, Junle Qu^1^***

Correspondence: jlqu@szu.edu.cn or weiyan@szu.edu.cn

^1^ Center for Biomedical Photonics & College of Physics and Optoelectronic Engineering, Key Laboratory of Optoelectronic Devices and Systems of Ministry of Education and Guangdong Province, Shenzhen University, Shenzhen 518060, China.

^2^ School of Chemistry and Chemical Engineering, Nanjing University, 163 Xianlin Road, Nanjing, 210023, China

^3^ Key Laboratory of Flexible Electronics (KLOFE) & Institute of Advanced Materials (IAM)
Nanjing Tech University (NanjingTech), 30 South Puzhu Road, Nanjing, 211816, China


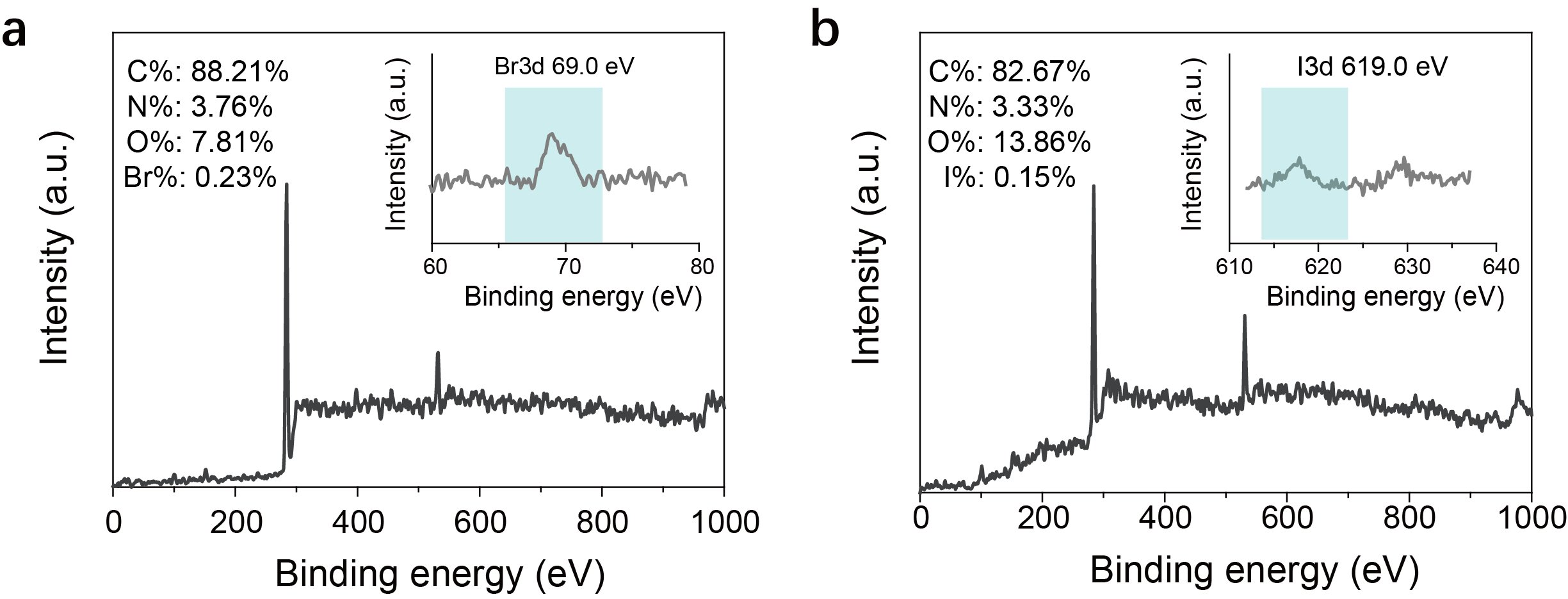


**Figure S1** XPS survey of CDs synthesized from Br/I doped precursors. **(a)** The XPS survey of p-bromanil-derived CDs. (Inserted: Br3d binding energy) **(b)** The XPS survey of p-bromanil-derived CDs. (Inserted: I3d binding energy)


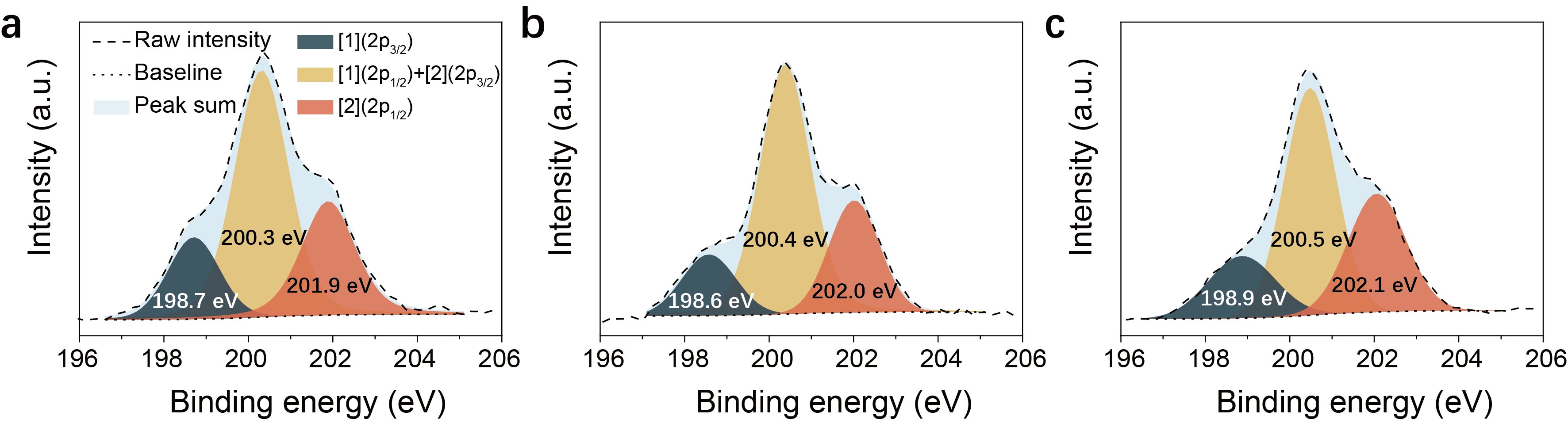


**Figure S2** Cl2p binding energy of ClCDs-1. **(a)**, ClCDs-2**(b)** and ClCDs-3**(c)**. In all three CDs, the Cl2p binding energy consist of three parts (two overlapping doublets). Here, component [1] referred to peripheral Cl binding to sp3 C, and [2] corresponds to Cl atoms binding with sp2C^s1, s2^.


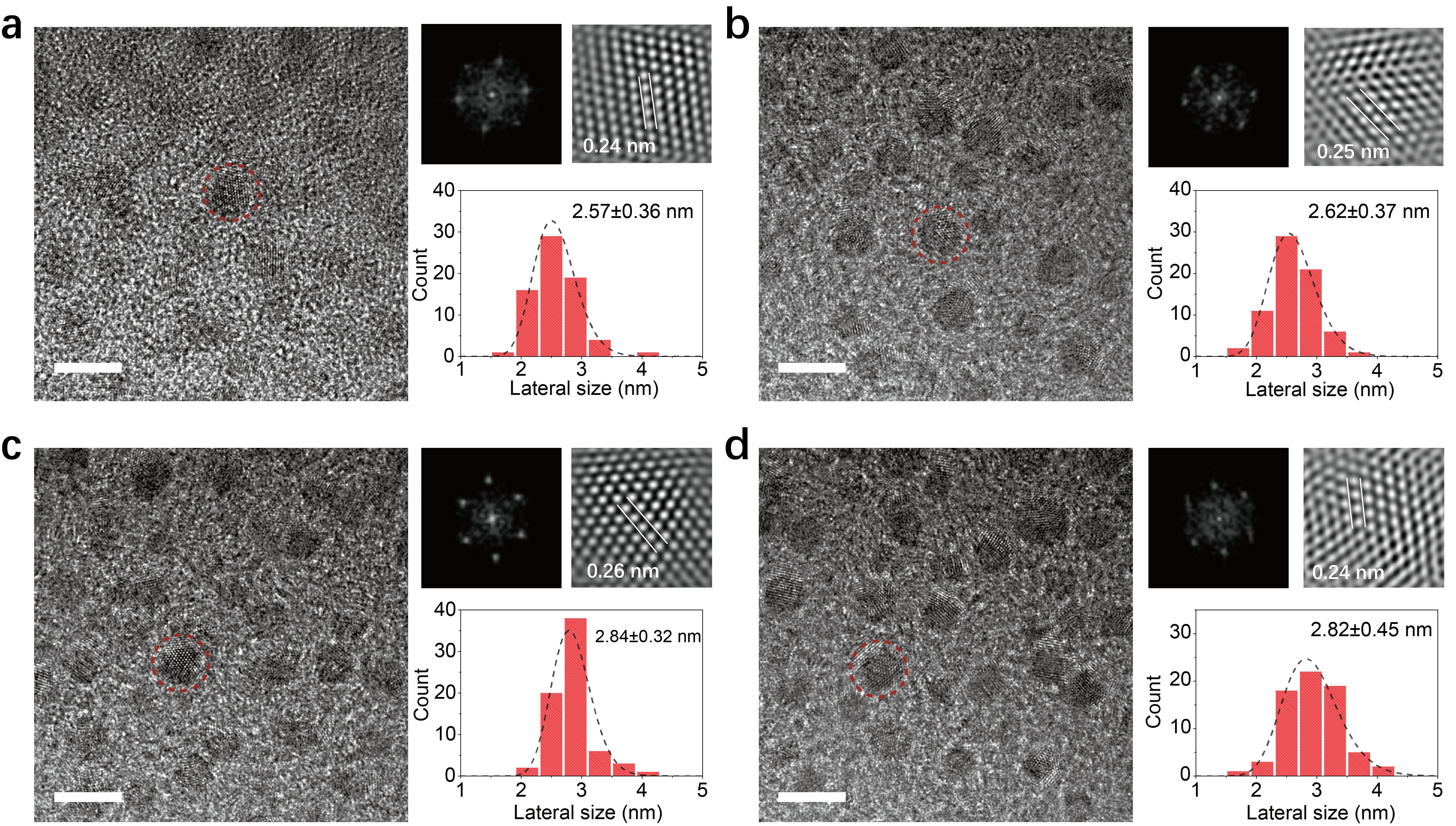


**Figure S3** TEM images of four CDs with different halogen contents (scale bar: 5 nm). **(a)** TEM images, FFT and inverse FFT results for CDs-0. **(b)** TEM images, FFT and inverse FFT results for ClCDs-1. **(c)** TEM images, FFT and inverse FFT results for ClCDs-2. **(d)** TEM images, FFT and inverse FFT results for ClCDs-3.


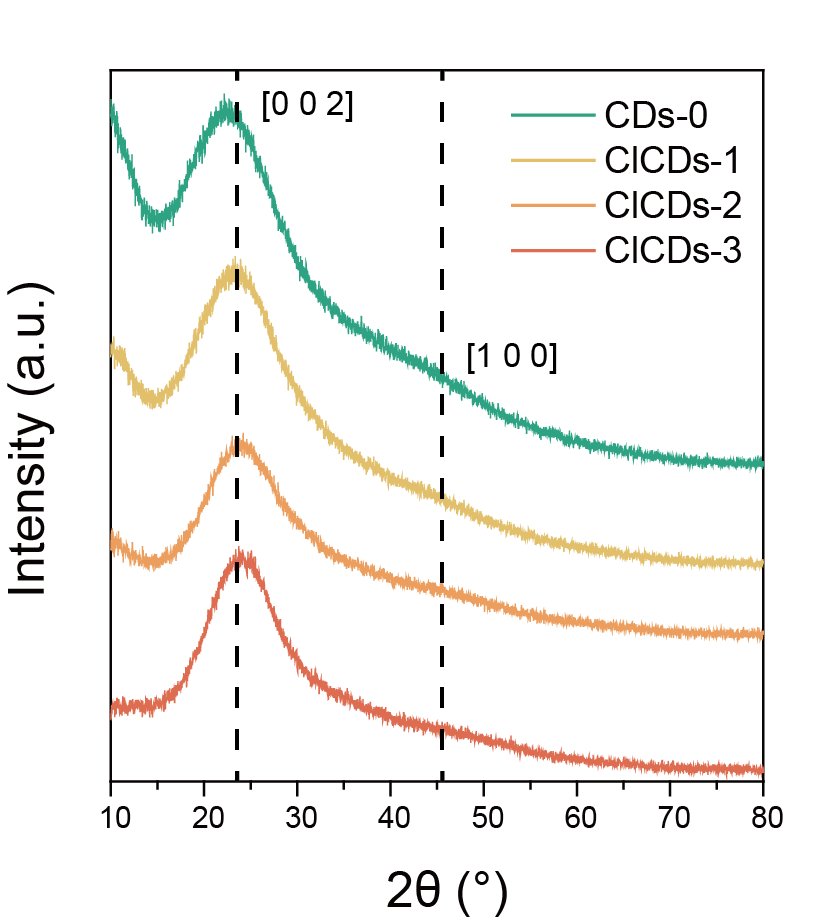


**Figure S4** The XRD patterns of all four CDs.


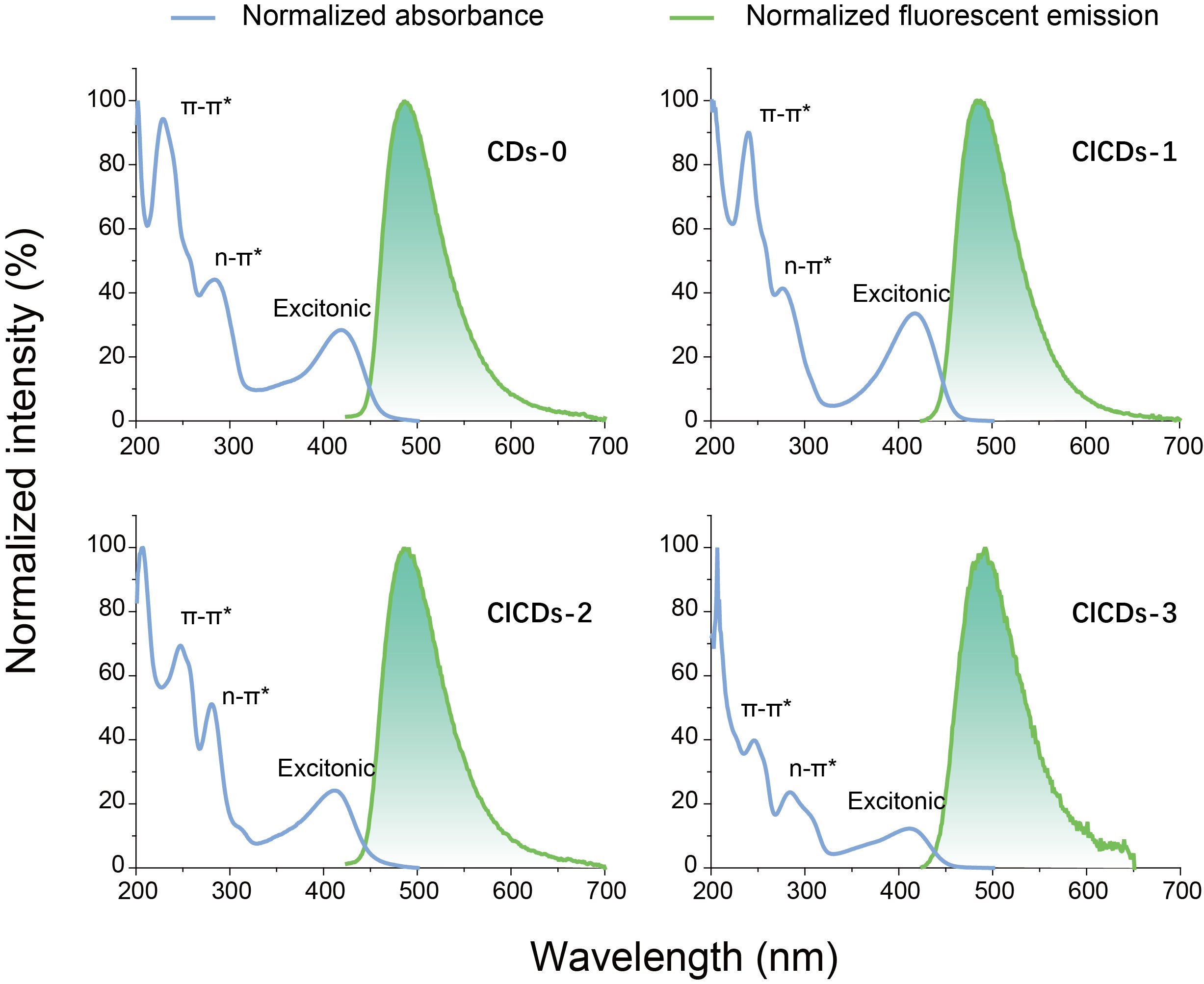


**Figure S5** The UV-Vis absorption and fluorescence emission of four CDs.


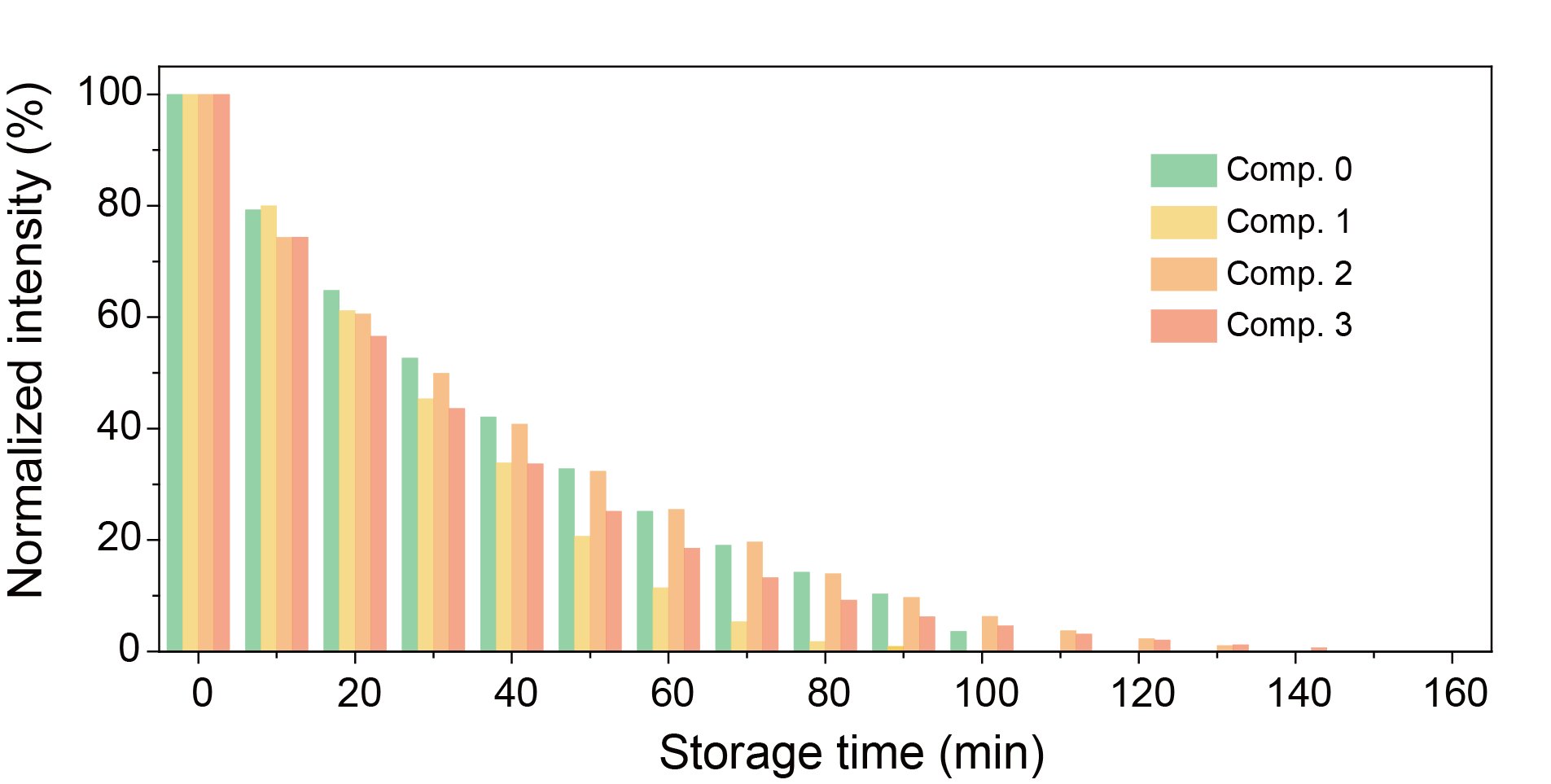


**Figure S6** Decrease of DURTP intensities in four CDs/PVP composites.


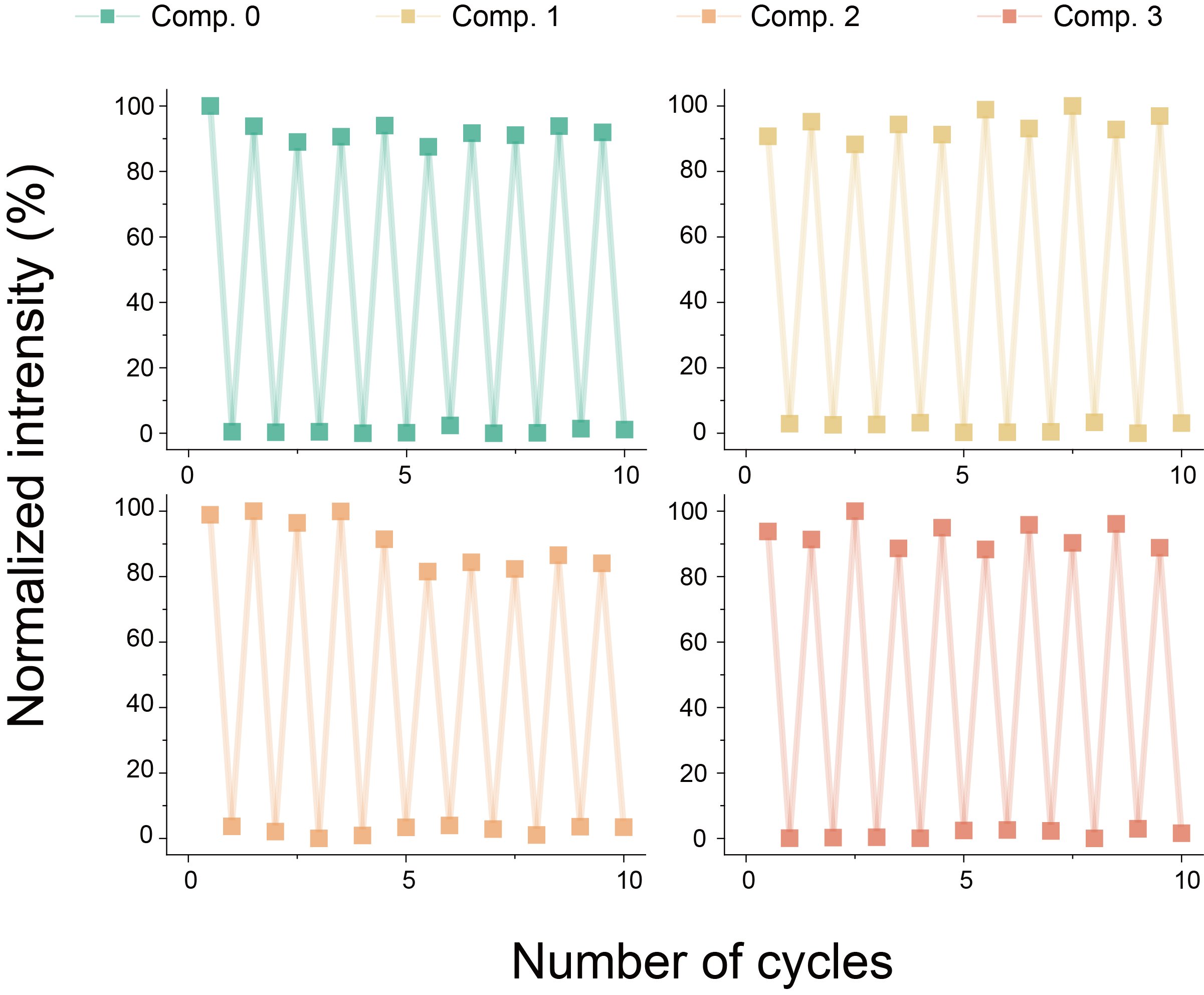


**Figure S7** The reversible on-off switch of DURTP in four CDs/PVP composites.


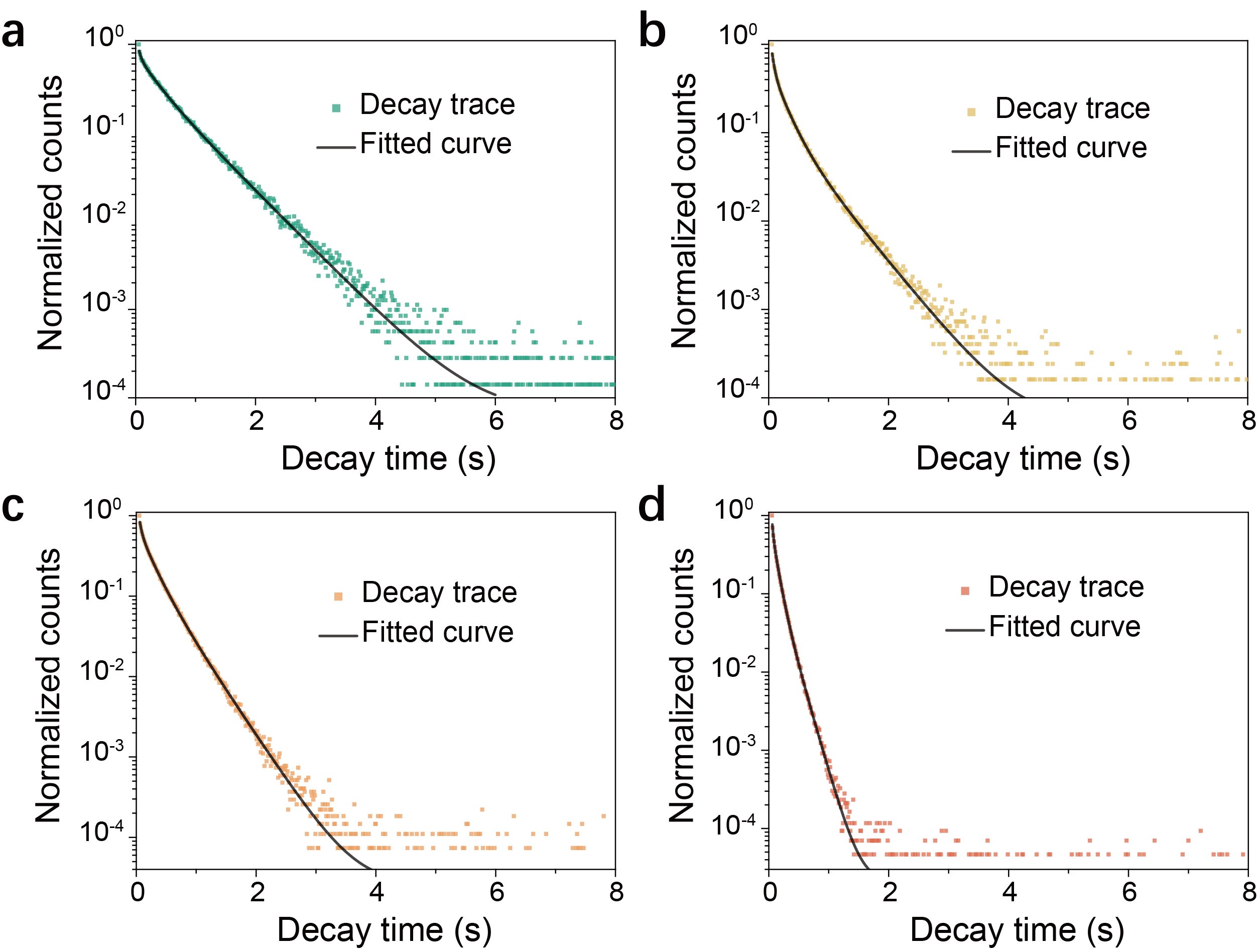


**Figure** **S8** The decay traces and fitted curves of the activated DURTP in composite 0**(a)**, composite 1**(b)**, composite 2**(c)** and composite 3**(d)**. (The lifetime decay trace was fitted with a triple-exponential decay model, where $\tau_{\mathrm{ave}}=\frac{{B_{1}\tau_{1}}^{2}+{B_{2}\tau_{2}}^{2}+{B_{3}\tau_{3}}^{2}}{B_{1}\tau_{1}+B_{2}\tau_{2}+B_{3}\tau_{3}}$)­


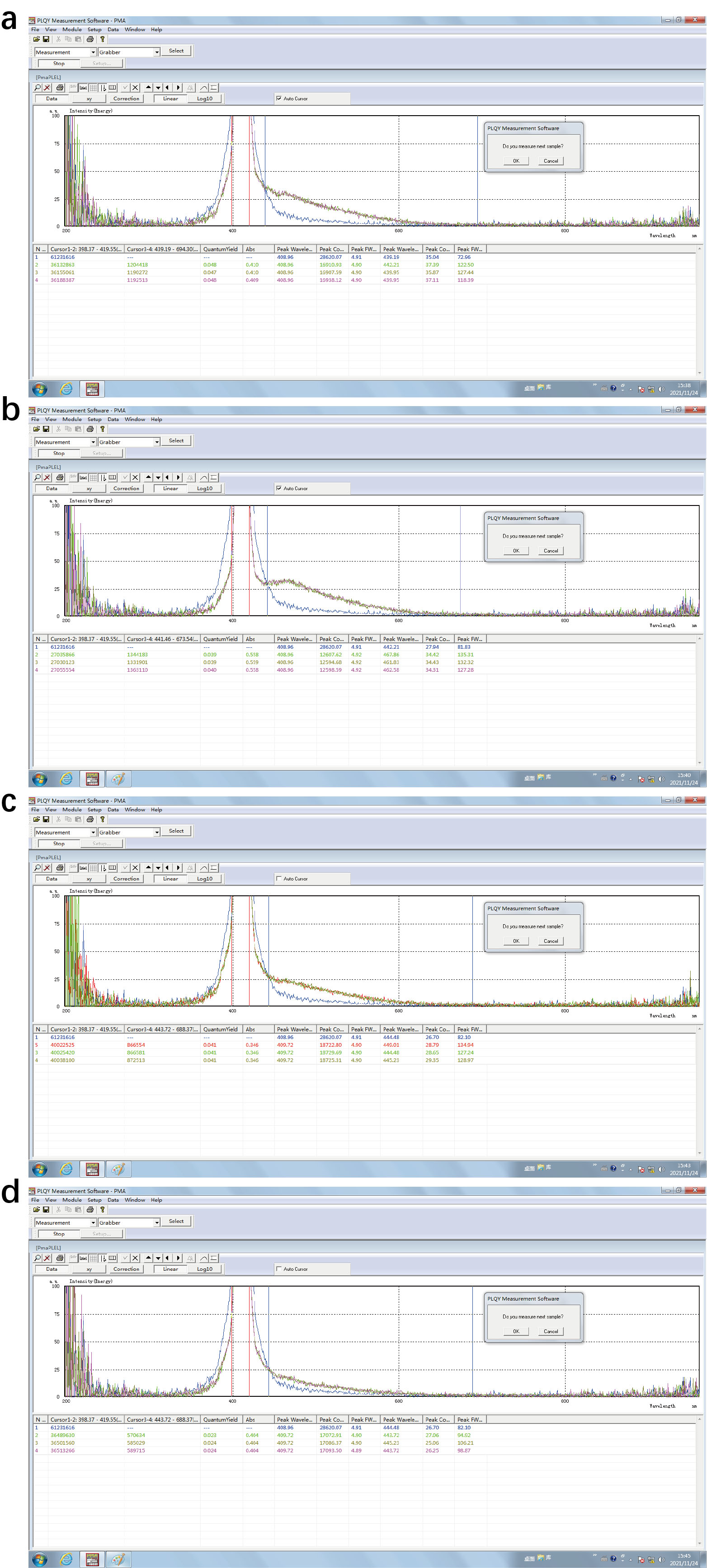


**Figure S9** The measurement of fluorescence quantum yields for composite 0 (**a**), composite 1 (**b**), composite 2 (**c**) and composite 3 (**d**).


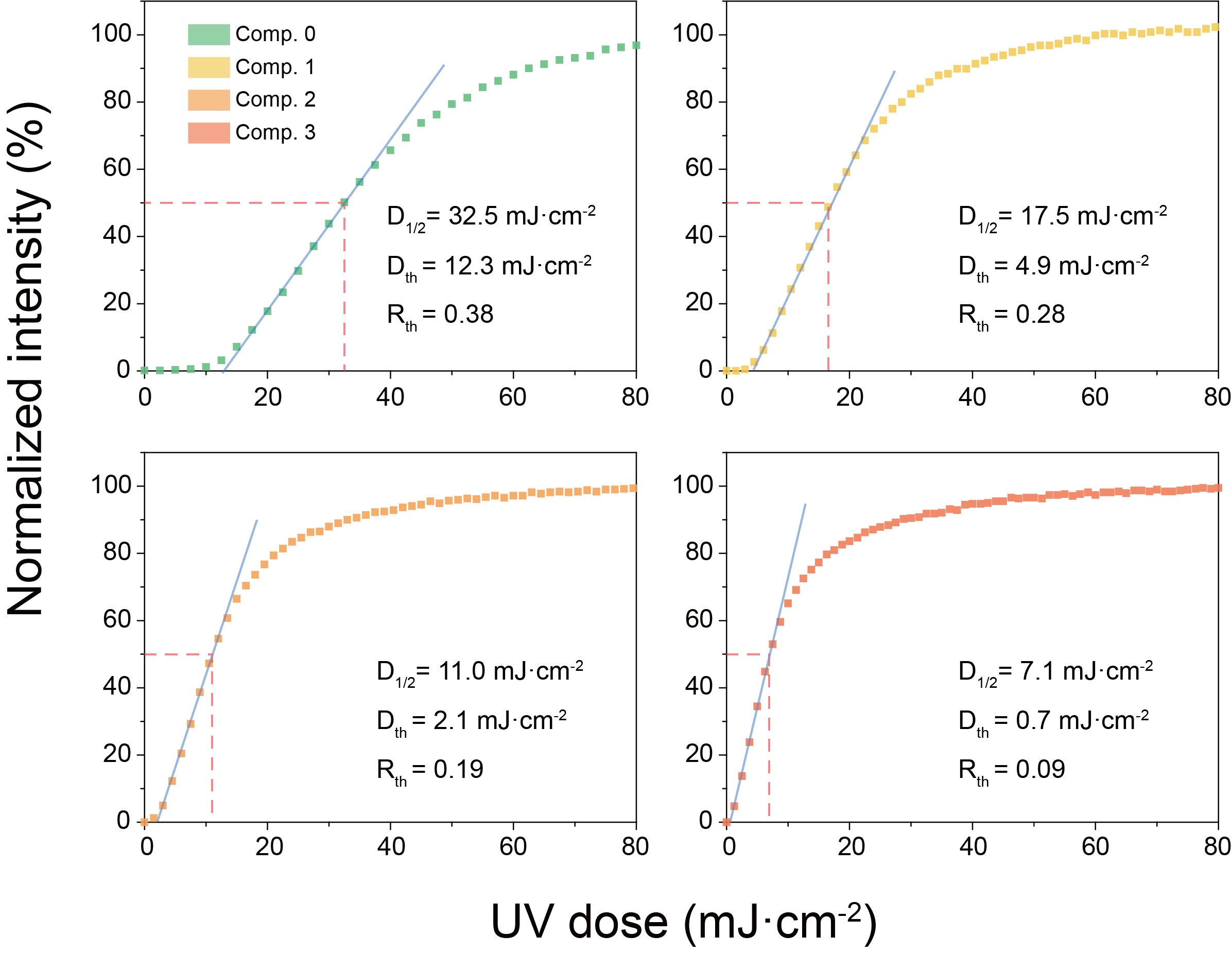


**Figure S10** The calculation of activation thresholds of four CDs/PVP composites. The UV dose was calculated by multiplying the UV intensity (0.1mW/cm^2^) with the activation time.

**
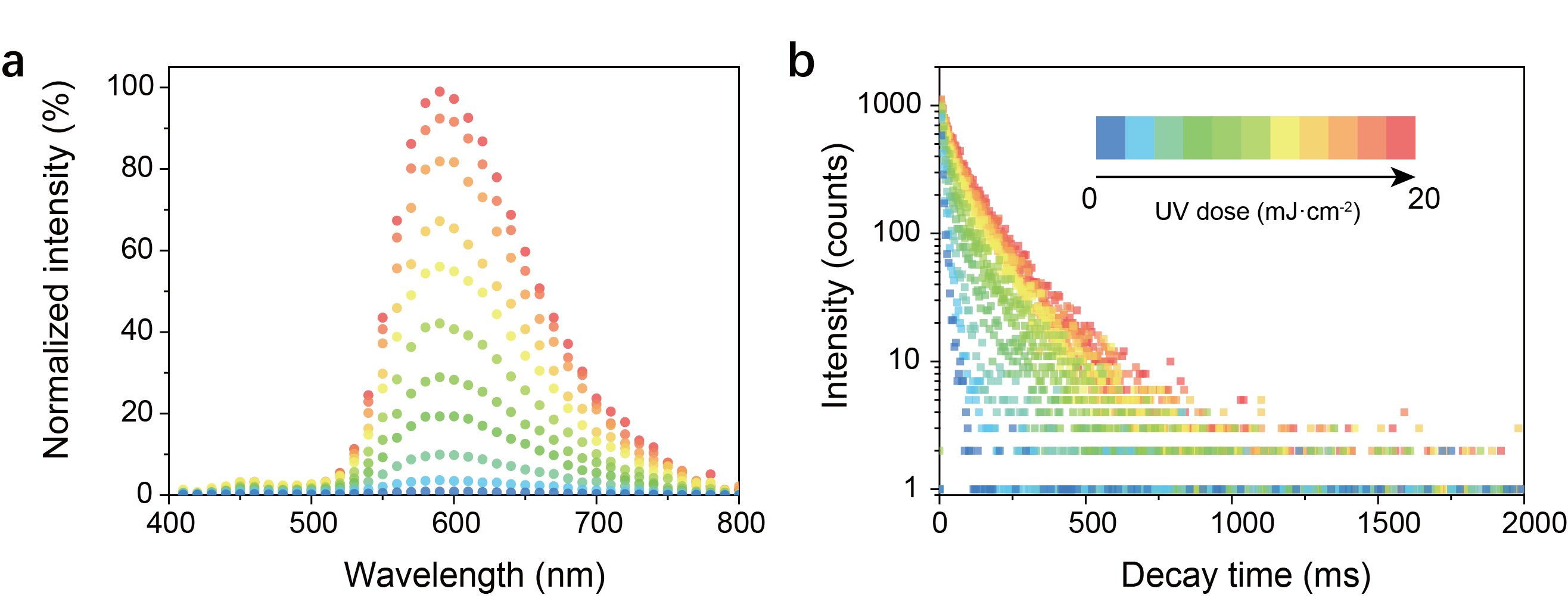
**

**Figure S11** The gradual change of lifetimes and DURTP intensities during the activation of composite 3.


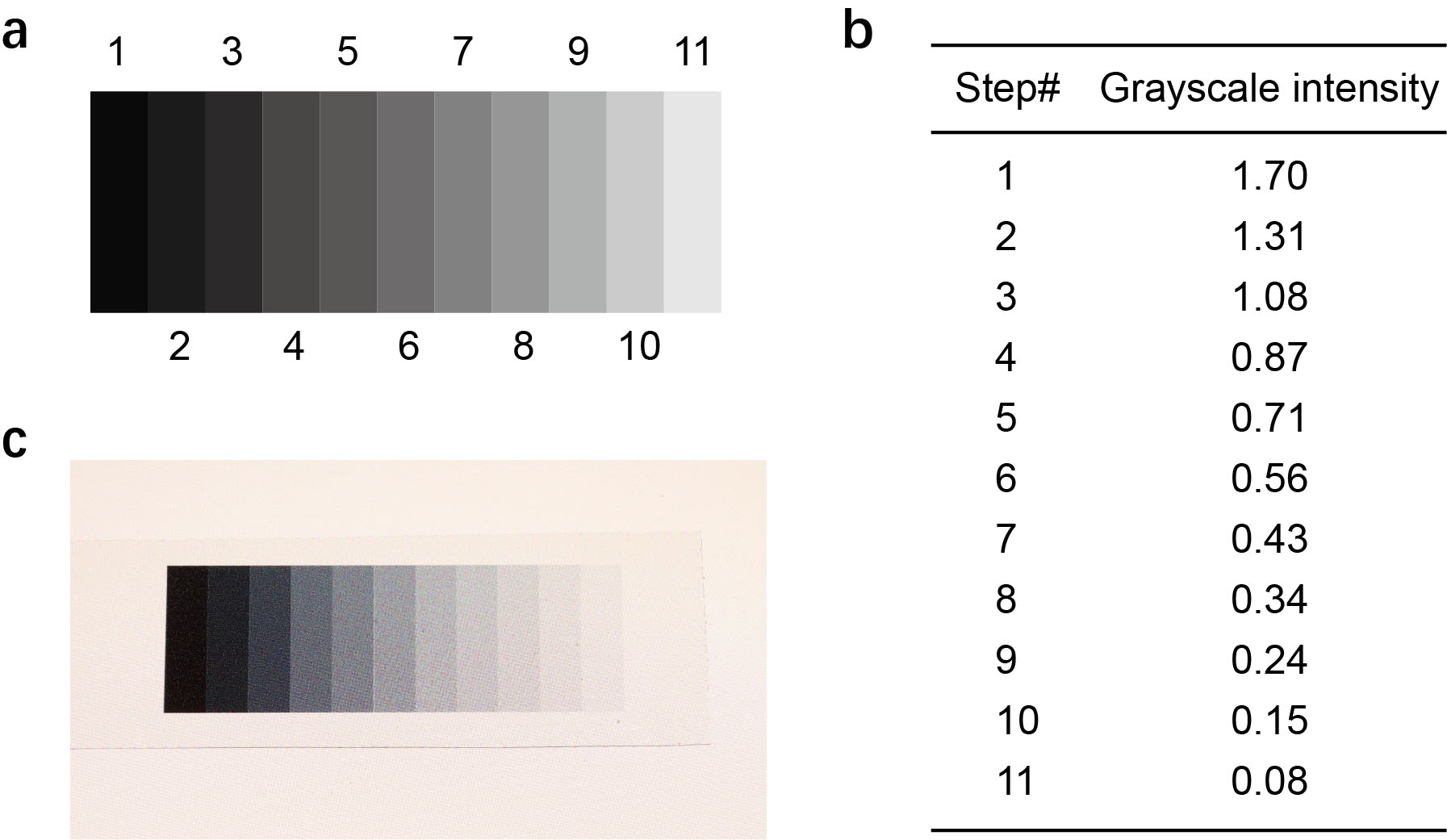


**Figure S12 (a)** The tailored ITE mask used for grayscale patterning. **(b)** The grayscale intensity values for each step presented in **a**. **(c)** Photograph of the printed mask.

**
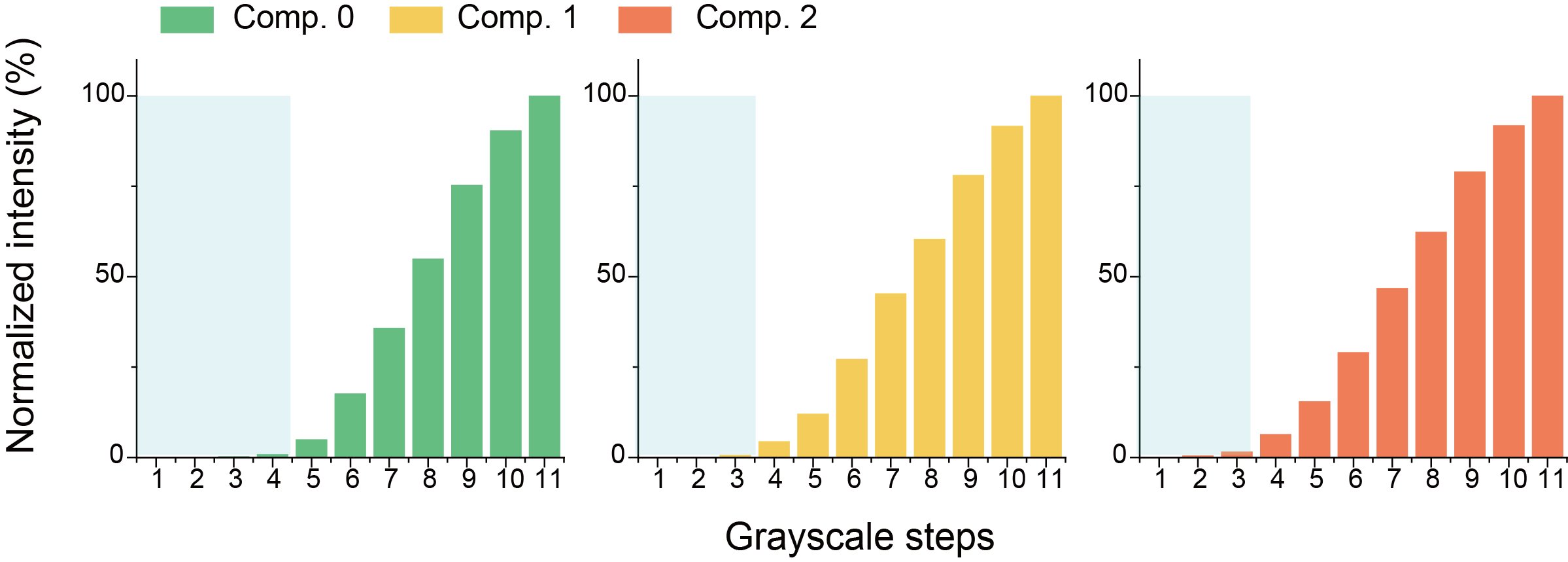
**

**Figure S13** The grayscale display capacity of composite 0~2 evaluated by the ITE grayscale step chart (calculated from the activation curve). Note that 3~4 steps are missed with the halogen-free composite 0.


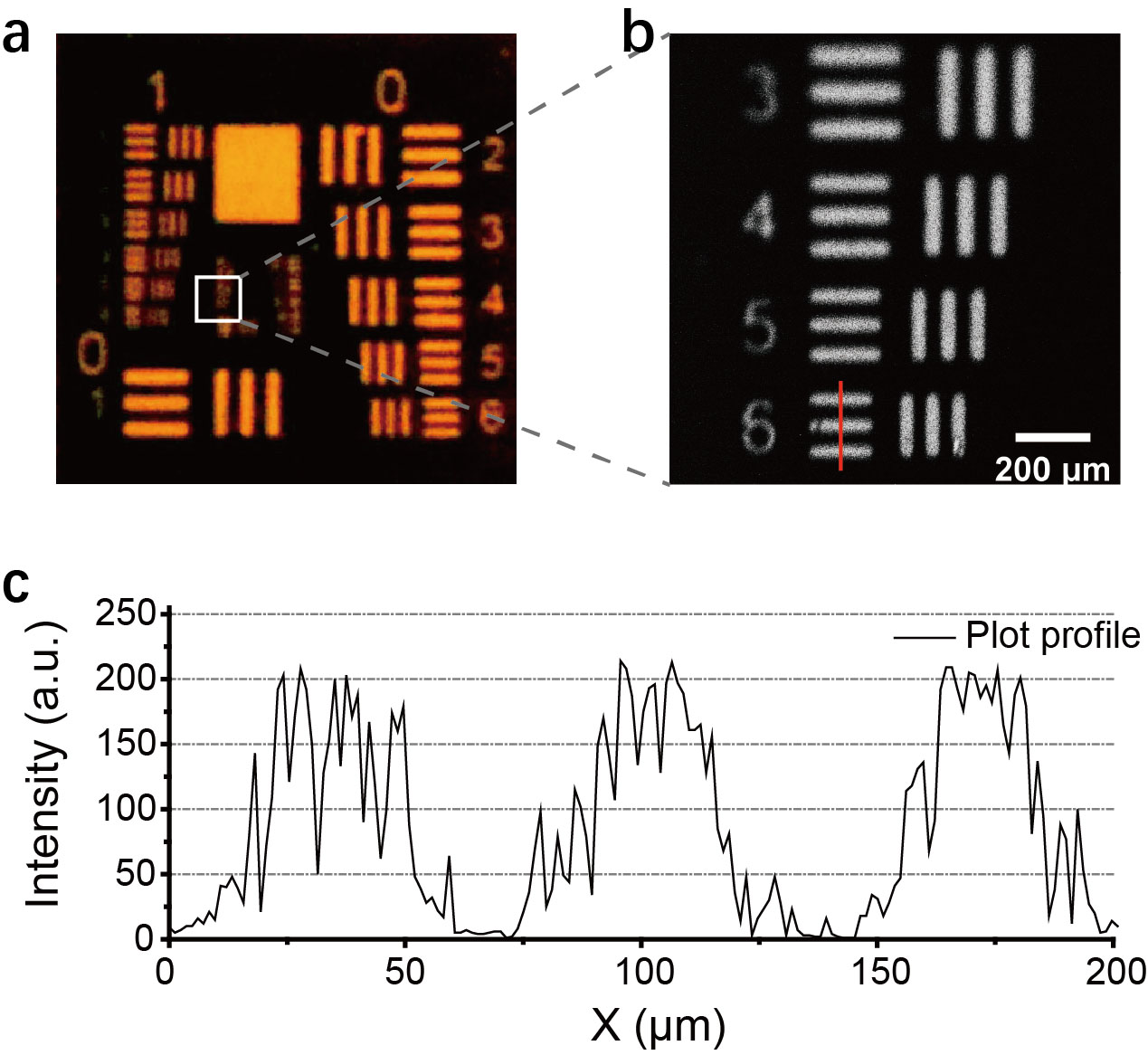


**Figure S14** The resolution of patterned CDs/PVP composite evaluated by a standard USAF-1951 target. **(a)** Delayed emission image of the USAF-1951 pattern captured with commercial camera. **(b)** The magnified image of group #3 by an industrial microscopic camera. **(c)** Line plot profile of the selected region (red line in **b**) across item #6, group #3 (Line width: 35.1 μm).


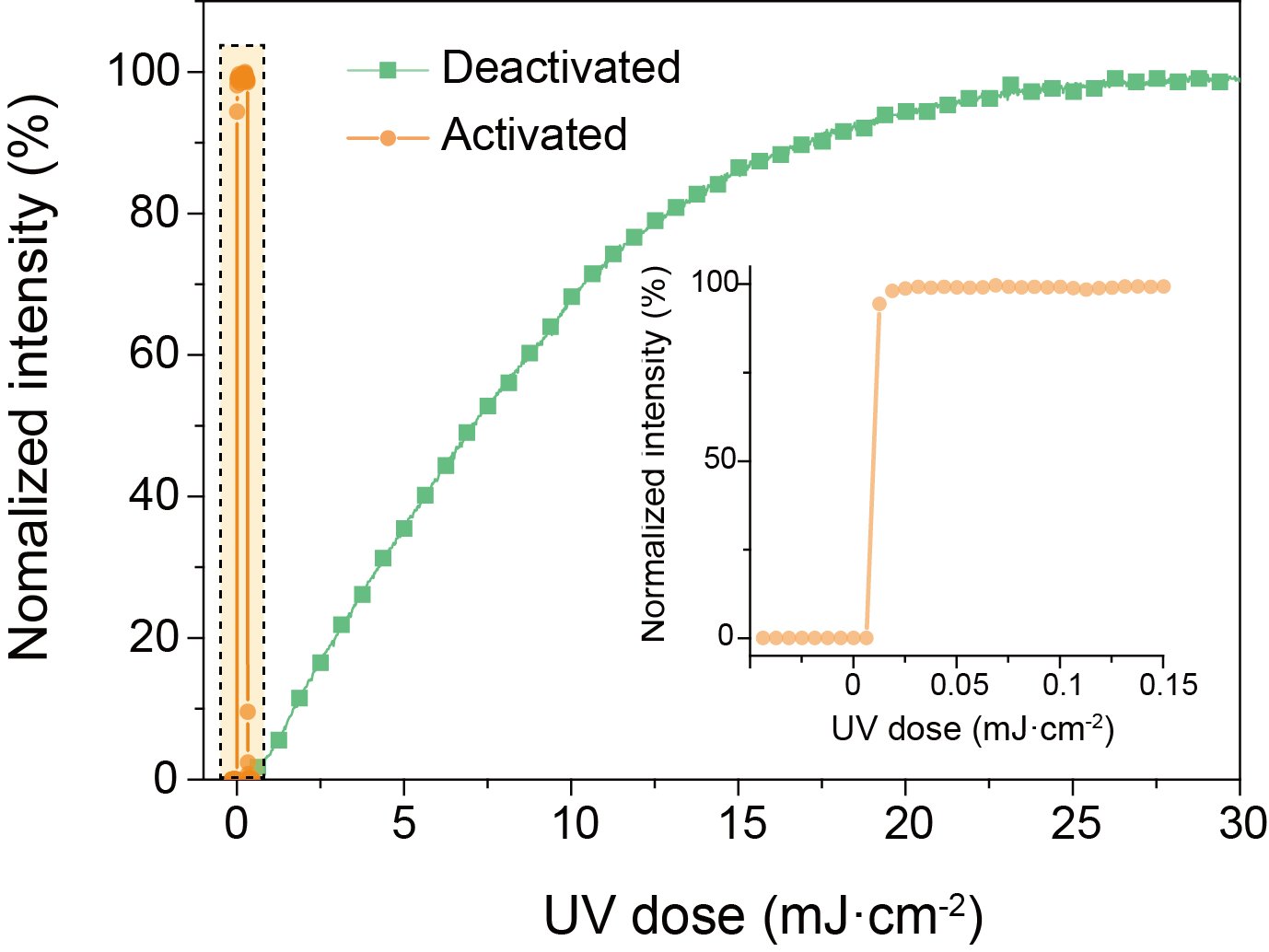


**Figure S15** The different excitation dynamics of deactivated and activated DURTP in composite 3.


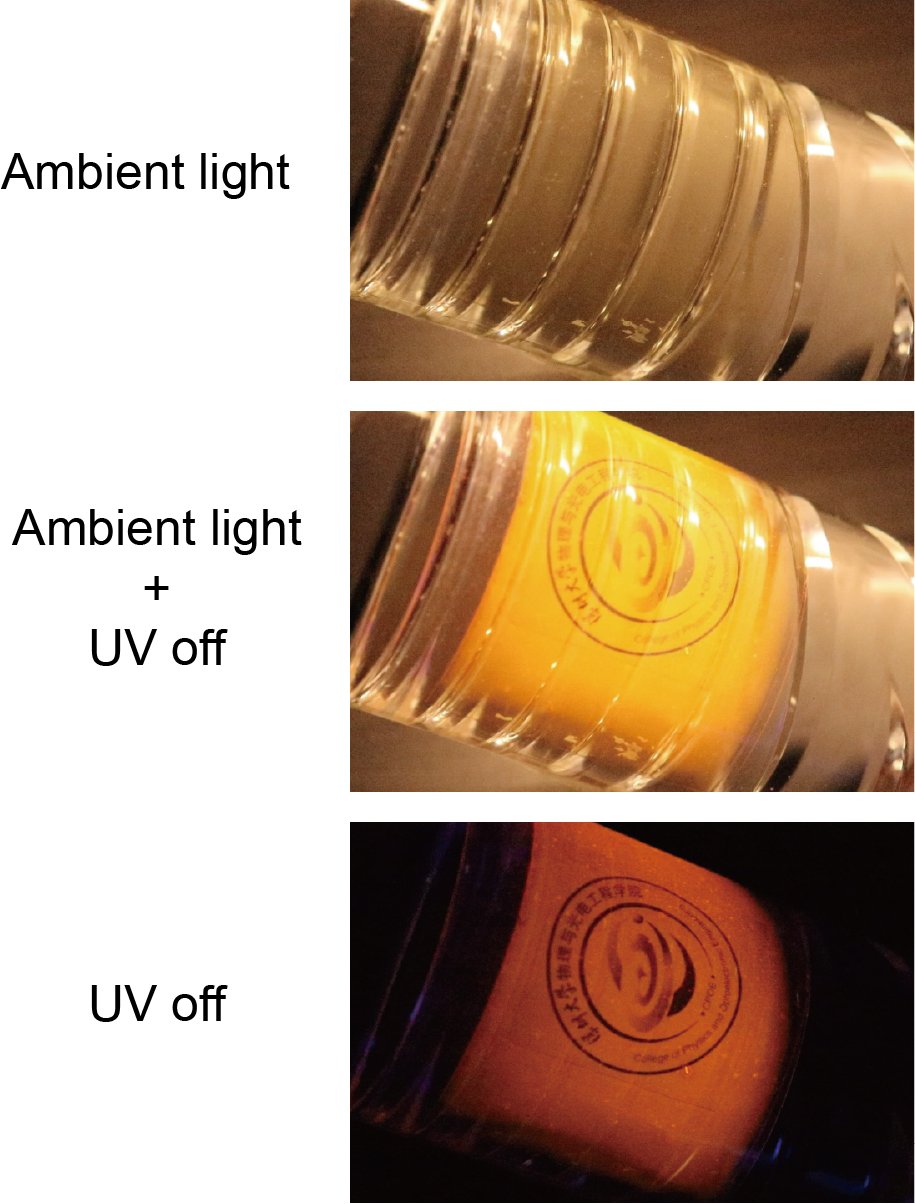


**Figure S16** The display of grayscale pattern on composite 3 attached to a curved surface.


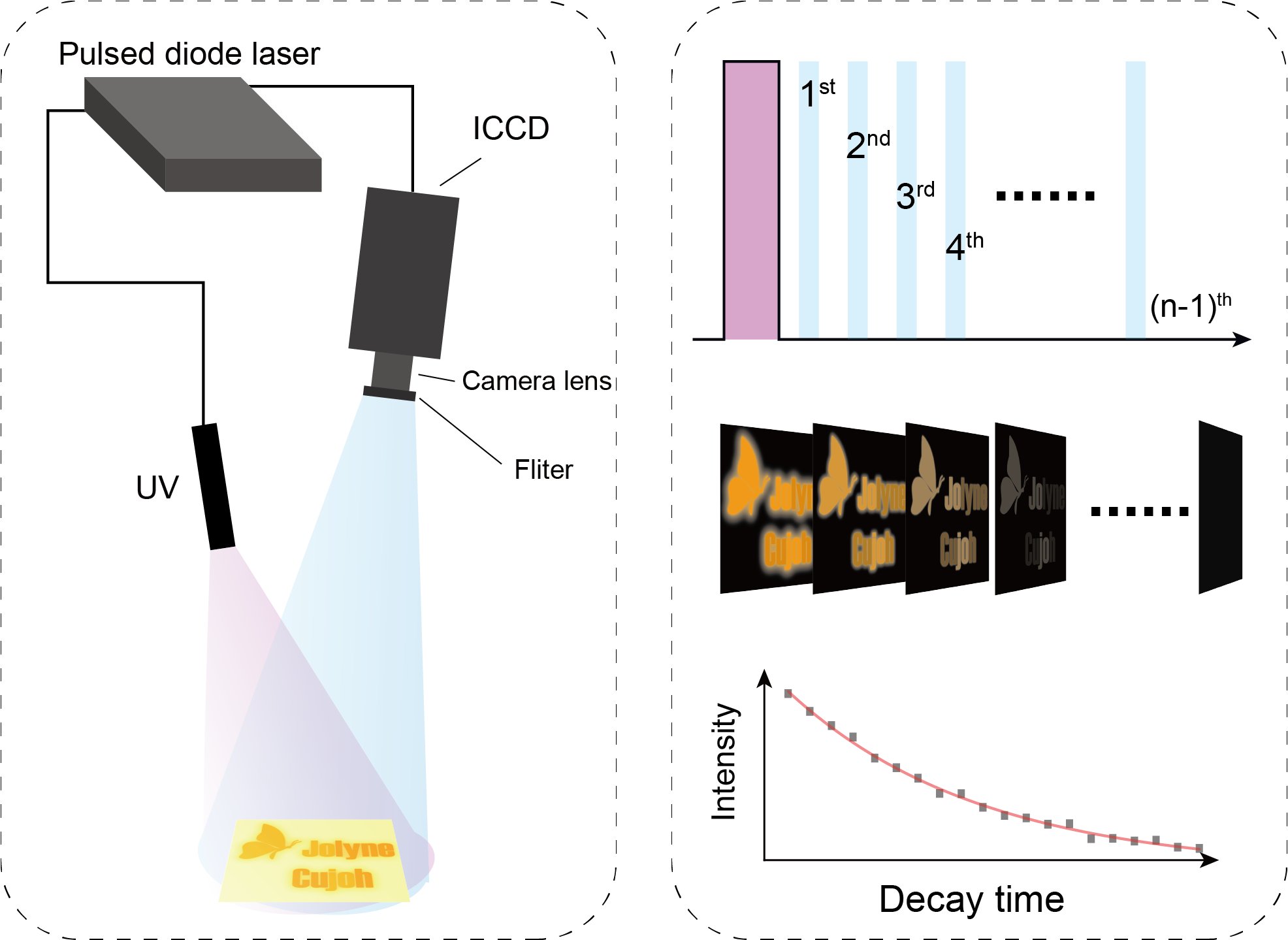


**Figure S17** Schematic illustration for the lifetime imaging setup.


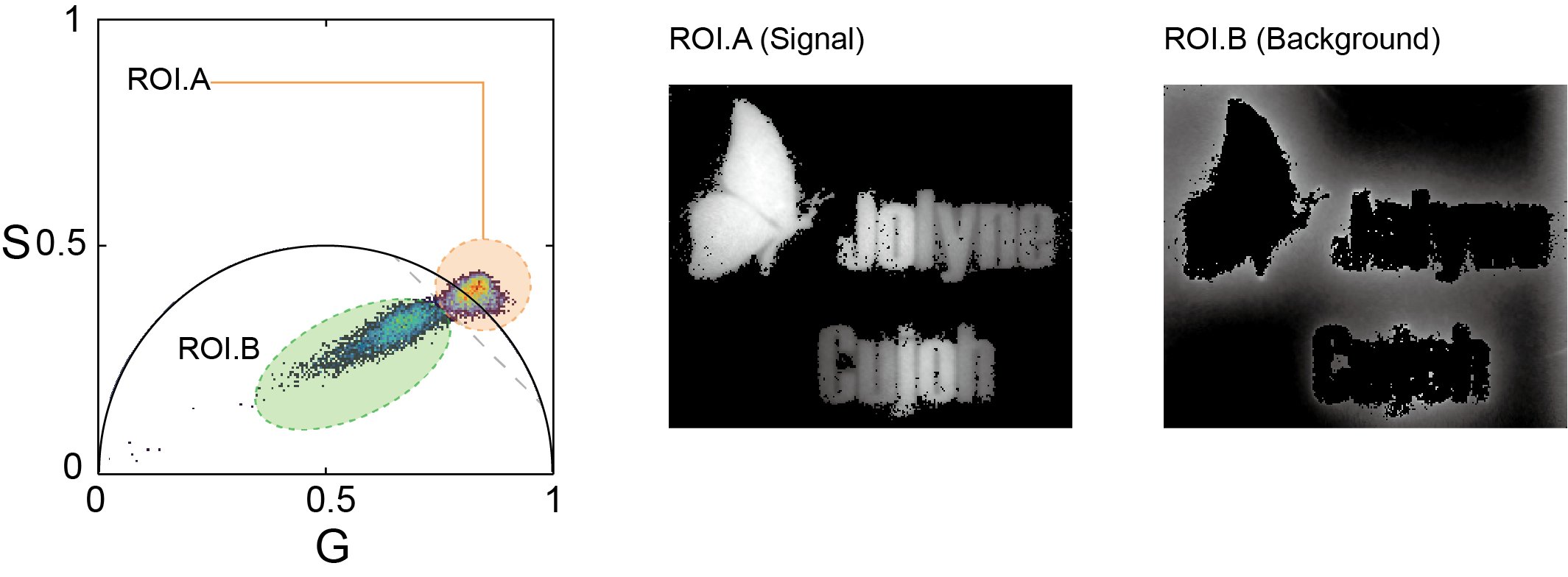


**Figure S18** The fast-denoising process by phasor analysis.

**Table S1** The assignment of FT-IR absorbance signals for four CDs

| **Type** | **Assignment** | **Wavenumber (cm^-1^)** | | | |
| --- | --- | --- | --- | --- | --- |
|  |  | **CDs-0** | **ClCDs-1** | **ClCDs-2** | **ClCDs-3** |
| N-H stretch/  O-H stretch | Amine/Hydroxyl | 3381 | 3411 | 3416 | 3411 |
| C-H stretch | Alkyl | 2921 | 2924 | 2922 | 2925 |
| C-H stretch | Alkyl | 2848 | 2850 | 2850 | 2852 |
| C=C /C=O/  C=N stretch  (Overlapped) | Double bonds | 1620 | 1648 | 1655 | 1656 |
| C=C stretch | Alkenyl | 1511 | 1489 | 1519 | 1521 |
| C-H bend | Alkyl | 1470 | \ | 1466 | 1456 |
| C-N stretch (C=C-N) | Amine | 1378 | 1381 | 1381 | 1384 |
| C-O stretch | Hydroxyl | 1230 | 1196 | 1190 | 1185 |
| C-N stretch (C-C-N)/C-Cl stretch (Overlapped) | Amine | 1097 | 1104 | 1107 | 1105 |

**Table S2** Lifetime fitting details of the activated DURTP in four CDs/PVP composites

| **Material** | ***τ*_1_ (ms)** | ***B*_1_** | ***τ*_2_(ms)** | ***B*_2_** | ***τ*_3_ (ms)** | ***B*_3_** | ***τ*_ave_ (ms)** | ***χ*^2^** |
| --- | --- | --- | --- | --- | --- | --- | --- | --- |
| Comp. 0 | 25.0 | 1319 | 179.0 | 1015 | 574.6 | 1217 | 476 | 1.028 |
| Comp. 1 | 41.7 | 3962 | 202.6 | 4462 | 522.9 | 1764 | 338 | 1.039 |
| Comp. 2 | 37.0 | 6463 | 193.1 | 9727 | 391.0 | 7270 | 299 | 1.050 |
| Comp. 3 | 17.8 | 10344 | 78.8 | 18692 | 165.1 | 7036 | 110 | 1.008 |

**Table S3** The calculation of phosphorescence quantum yields of four CDs/PVP composites

| **Composites** | **Fluorescence quantum yield** | ***I*_Phos._/*I*_Fluo._** | **Phosphorescence quantum yield** |
| --- | --- | --- | --- |
| Composite 0 | 4.7% | 0.20 | 0.94% |
| Composite 1 | 4.0% | 0.43 | 1.60% |
| Composite 2 | 4.1% | 0.47 | 1.93% |
| Composite 3 | 2.4% | 1.22 | 2.93% |

**Movie S1 caption:** The fully programmable writing, reading, burn-after-read and erasing of different patterns with grayscale DURTP.

**References:**

s1 Li B, Zhou L, Wu D, Peng H, Yan K, Zhou Y *et al.* Photochemical Chlorination of Graphene. *ACS Nano* 2011; **5**: 5957–5961.

s2 Papirer E, Lacroix R, Donnet J, Nanse G, Fioux P. Xps Study of the Halogenation of Carbon Black-Part 2 . Chlorination. *Carbon* 1994; **33**: 63–72.
